# Adults and Children in Low-Income Households That Participate in Cost-Offset Community Supported Agriculture Have High Fruit and Vegetable Consumption

**DOI:** 10.3390/nu9070726

**Published:** 2017-07-08

**Authors:** Karla L. Hanson, Jane Kolodinsky, Weiwei Wang, Emily H. Morgan, Stephanie B. Jilcott Pitts, Alice S. Ammerman, Marilyn Sitaker, Rebecca A. Seguin

**Affiliations:** 1 Division of Nutritional Sciences, Cornell University, Ithaca, NY 14853, USA; ehm72@cornell.edu (E.H.M.); rs946@cornell.edu (R.A.S.); 2 Department of Community Development and Applied Economics, University of Vermont, Burlington, VT 05405, USA; jane.kolodinsky@uvm.edu (J.K.); weiwei.wang@uvm.edu (W.W.); 3 Department of Public Health, Brody School of Medicine, East Carolina University, Greenville, NC 27834, USA; jilcotts@ecu.edu; 4 Gillings School of Global Public Health and School of Medicine, University of North Carolina at Chapel Hill, Chapel Hill, NC 27599, USA; alice_ammerman@unc.edu; 5 The Evergreen State College, Ecological Agriculture and Food System, Olympia, WA 98505, USA; msitaker@gmail.com

**Keywords:** food access, food insecurity, dietary quality, community supported agriculture, local foods

## Abstract

This paper examines fruit and vegetable intake (FVI) in low-income households that participated in a cost-offset (CO), or 50% subsidized, community-supported agriculture (CSA) program. CSA customers paid farms upfront for a share of the harvest, and received produce weekly throughout the growing season. A cohort of adults and children 2–12 y in a summer CO-CSA were surveyed online twice: August 2015 (*n* = 41) and February 2016 (*n* = 23). FVI was measured by the National Cancer Institute’s (NCI) Fruit and Vegetable Screener (FVS) and an inventory of locally grown fruits and vegetables. FVI relative to United States (US) recommendations and averages, and across seasons, were tested with non-parametric tests and paired *t*-tests (*p* < 0.05). Both adults and children in the CO-CSA had higher FVI than the US averages, and more often met recommendations for vegetables. Some summer fruits and vegetables were more often eaten when locally in-season. The CO-CSA model warrants further examination as an avenue for improving vegetable consumption among adults and children in low-income households. However, causality between CO-CSA participation and FVI cannot be inferred, as CO-CSA participants may be positive deviants with respect to FVI. A multi-state randomized controlled trial is currently underway to evaluate impacts of CO-CSAs on FVI and related outcomes.

## 1. Introduction

Eating a diet rich in fruits and vegetables is associated with the prevention of poor health outcomes in adults [1,2,3]. Although evidence is currently inadequate to substantiate a link between fruit and vegetable intake (FVI) and obesity or other health outcomes among children [4,5], children’s eating habits nevertheless remain important because they persist into adulthood and contribute to adult diet-related disease [6]. The 2010 Dietary Guidelines for Americans (DGAs) recommend consumption of two to six and one-half cups of fruits and vegetables per day depending upon sex, age, and level of physical activity [7]. However, the average American’s total FVI is less than half of the recommended amount [8,9]. FVI is lower among individuals with low socioeconomic status [10,11] and food insecurity [12] than among their higher-income and more food-secure counterparts. Children’s dietary quality is known to be associated with home availability and accessibility of healthy foods such as fruits and vegetables [4,13]. Low income communities have lesser availability of quality fresh fruits and vegetables in their local food outlets [14,15,16,17], higher cost for produce [11,14,16], and poorer physical access to stores that stock fresh produce [14,15], all of which may hinder FVI.

Recent research suggests that shopping at farmers’ markets is positively associated with FVI [18,19,20], and that programs that facilitate access to farmers’ markets for families with low-incomes significantly increase self-reported vegetable consumption [21,22,23,24]. None of these studies, however, measured FVI among children. The United States Department of Agriculture (USDA) recently awarded more than $30 million to test innovative ideas about how to increase the purchase of fruits and vegetables by low-income consumers, and particularly to connect these consumers with agricultural producers and locally-grown products [25].

### 1.1. Community Supported Agriculture

Community supported agriculture (CSA) is one mechanism that may improve access to fresh produce for low-income households. In CSAs, customers pay for a ‘share’ of produce before the growing season begins, and farmers provide fruits and vegetables weekly throughout the season [23,26,27,28,29,30,31,32,33]. In 2015, there were 5789 CSA operations in the US [34]. CSAs can improve access to fresh produce because they are located within the communities they serve [27,35], and often provide higher quality produce at a lower price than grocery stores [23,28,36]. In cross-sectional surveys, CSA members commonly report consuming a larger quantity and broader variety of vegetables after joining a CSA [27,37,38], and longitudinal studies suggest some impact of CSAs on at least some measures of FVI [32,39,40,41]. However, most current CSA members are from middle- to high-income households [28,36,42,43]. Affordability is a significant factor in CSA participation and retention [23,28,33,44], and the cost of CSAs may inhibit participation among low-income households in particular. A subsidized or cost-offset CSA (CO-CSA) that offers flexible payment schedules and discounted CSA shares can reduce cost as a barrier to participation among low-income households [45]. However, there is currently little evidence as to whether the high levels of FVI observed among higher-income adult CSA participants will be similarly high among lower-income adults and children who join CSAs.

### 1.2. Objectives

In this study, we compared FVI for adults and children from households that participate in a CO-CSA with US dietary recommendations and averages, and hypothesized that CO-CSA participants would have higher FVI. We also contrasted total FVI during the summer CO-CSA season and in the winter (without the CO-CSA) and expected that participants would have higher FVI during the summer CO-CSA than in the winter. Third, we examined seasonal variation in the varieties of fruits and vegetables consumed by CO-CSA participants, and hypothesized that produce varieties that were in-season would be consumed more frequently than those out of season. Examining FVI among adults and children that participate in a CO-CSA will add to our understanding of the potential for CSAs to support positive dietary change for members of low-income and food insecure households.

## 2. Materials and Methods 

Design and Setting. This paper reports on a longitudinal survey of adults and children who participated in a CO-CSA program operated by the Northeast Organic Farming Association of Vermont (NOFA-VT) in the US [46]. The NOFA-VT CO-CSA provides 25% of the CSA share price to farms in an effort to link limited-income individuals with farmers seeking local markets for their produce. Farms must match that 25% subsidy through donations from shareholders, fundraising efforts, or local community contributions. CO-CSA recipients pay the remaining 50% of the cost of the share [46]. NOFA-VT distributes CO-CSA informational brochures via farms and community organizations throughout the state, and brochures and applications also are available on-line [46]. Eligibility rules require only that applicants self-report household income ≤185% of the federal poverty level given family size ($44,467 for a family of four at the time of this study) [47]. Applications typically exceed available subsidies, which are provided on a first-come, first-served basis to participants who have identified agreeable farms [46]. Summer CSAs generally operate from June through October in VT.

A longitudinal survey was conducted in the summer (August 2015) and again the following winter (February 2016). All participants were already enrolled in the NOFA-VT CO-CSA at the summer survey. Participants in the winter survey reported they were not participating in any type of CSA. Institutional Review Boards at Cornell University and the University of Vermont approved the study protocol, and consent was obtained electronically. 

Participants. Names and contact information for all 179 low-income NOFA-VT CO-CSA applicants for summer 2015 were obtained, and households that did not report a child aged 2–12 years (*n* = 85), did not obtain a CO-CSA (*n* = 12), or who did not provide an email address (*n* = 1) were excluded. The remaining 81 households were invited to the summer online survey. Summer survey participants were subsequently invited to complete the winter survey. Participants were compensated $25 for each survey they completed.

Variables and Data Sources. Dietary data were obtained for one adult and one randomly-selected ‘focal’ child aged 2–12 years in each household. Three types of dietary outcome variables were examined: FVI (cups), indicators of meeting or exceeding recommendations, and frequencies of consuming particular fruits and vegetables (times per month). The child’s dietary data were generally reported by the parent who prepared most of the child’s meals, reporting either with the child (35%) or alone (58%). FVI was assessed with the NCI All-Day Fruit and Vegetable Screener (FVS). The validated FVS collected the frequency and usual portion size for nine fruit and vegetable components (100% juice, fruit, lettuce salad, fried potatoes, other white potatoes, cooked dried beans, other vegetables, tomato sauce, and vegetable soup) in the past month [48]. We formatted on-line questions about usual portion size to include corresponding photographs in an effort to reduce reporting bias [49]. The product of frequency and portion responses was converted into daily 2005 MyPyramid Cup Equivalents for each FVS component, and summed to measure total FVI. Age- and sex-specific calorie, fruit, and vegetable DGA recommendations [7] were identified for each participant, assuming a sedentary level of physical activity. Two dichotomous variables indicated whether a participant met their recommendation (fruit or vegetable), or not.

For 16 varieties of vegetables and 12 fruits that were locally grown and only seasonally available [50,51], frequency of consumption was recorded by an intake questionnaire with eight ordinal response choices (from never to 3+ times per day), and converted into times/month. All fruits and most vegetables were considered ‘in-season’ during summer data collection; whereas celeriac, parsnips and sweet potatoes were considered ‘in-season’ during winter data collection.

Household, respondent, and child characteristics were collected as part of the summer survey. Participants provided a household roster from which number of adults and children, sex and age of the respondent, and sex and age of the focal children were recorded. Household food security was classified by the 6-item short form of the USDA Food Security Survey Module (FSSM), with a 30-day reference period [52]. Two questions assessed participation in the Supplemental Nutrition Assistance Program (SNAP) and the Special Supplemental Food Program for Women, Infants, and Children (WIC) in the past month [53]. Respondents reported how difficult it was to financially afford, and to physically access, acceptable quality fruits and vegetables (FV) with five ordinal responses that were subsequently collapsed into ‘difficult (extremely or relatively)’ or not. Respondent ethnicity and education were also recorded.

Statistical Methods. Sample characteristics were summarized with frequencies and percentages using SPSS version 23 software [54]. US median FVI for adults [8] and for children [9] were weighted to reflect the sex and age composition of this sample. Sample estimates of median FVI were compared to the weighted US parameters using Wilcoxon signed rank tests. US percentages meeting fruit and vegetable recommendations [55] were also weighted, and sample estimates of percentages meeting fruit and vegetable recommendations were compared to the weighted US parameters using non-parametric binomial tests. Sample median summer total FVI was compared to median winter total FVI using Wilcoxon signed rank tests. Mean frequency of consumption for each fruit and vegetable variety was contrasted when locally in-season and out-of-season using two-tailed paired t-tests. 

## 3. Results

Forty-one CO-CSA participants responded to the summer survey (50.6%). Most participant households included two adults (71.0%) and multiple children (75.6%; Table 1). More than half of CO-CSA participants had low or very low food security (53.8%), received SNAP benefits (51.3%), and considered it difficult to financially afford acceptable quality fruits and vegetables (53.8%). Few CO-CSA participants (7.7%) reported having difficulty physically accessing acceptable quality fruits and vegetables. Most respondents were white (87.5%), women (87.2%), and two-thirds had graduated from college (69.2%).

Both adults and children in CO-CSA households consumed more fruits and vegetables than the US median (*p* < 0.01; Table 2). Adults and children in the CO-CSA also were more likely to meet DGA recommendations for vegetables (but not fruits) than typical (*p* < 0.01). Notably, 81.6% of adults and 82.9% of children in CO-CSA participant households met DGA recommendations for vegetable intake.

Of the 41 summer survey respondents, 23 also completed a winter survey (56.1%). Median total FVI in the summer and winter did not significantly differ for adults (6.44 vs. 6.39 cups/day) or children (4.46 vs. 3.89 cups/day; data not shown). However, patterns of seasonality emerged in the types of fruits and vegetables consumed (Table 3). When locally in-season, both adults and children more often ate summer vegetables (cucumbers, summer squash, and tomatoes) and fruits (blueberries, cherries, and peaches). Adults also more often ate other summer vegetables when locally in-season (eggplant and peas), whereas children more often ate other summer fruits when in-season (plums, raspberries). None of the winter varieties about which we asked (celeriac, parsnips and sweet potatoes) were consumed more often when locally in-season (winter) than when out-of-season. 

## 4. Discussion

CO-CSA participants and their children reported total FVI greater than the US average, and more often met recommendations for vegetable consumption than the US population. They also more often consumed some summer vegetables and fruits when locally in-season than when out-of-season. Our findings are consistent with prior studies that found adult CSA members consumed a larger quantity of fruits and vegetables than their non-CSA counterparts [27,37,38,39,40,41], and add important evidence that children in CO-CSA households also have higher FVI. Furthermore, all households in our sample had low incomes, and more than half were food insecure, which are circumstances known to increase risk for inadequate FVI [10,11,12,14,15,16]. In addition, less than eight percent of this sample reported having difficulty physically accessing fruits and vegetables, in contrast to the extensive barriers reported in low-income communities.[15,57] Together, these findings suggest that access barriers were rare in this sample, and that CO-CSA participation may have positively contributed to fruit and vegetable access and consumption.

While these data suggest higher-than-expected FVI among low-income CO-CSA participants, we cannot infer causality between CO-CSA participation and FVI from these observational data. Although all the CO-CSA participants had low-incomes, the majority of respondents were highly educated, which suggests that they were atypical of the US population with low-incomes. Passive recruitment approaches may have contributed to the selection of motivated and atypically educated participants. Education is known to be positively associated with healthy dietary behaviors such as vegetable consumption, which further introduces selection bias. Consistently high FVI reported in summer and winter (time points with and without the CO-CSA) supports the likelihood that selection bias contributed, at least in part, to the high levels of FVI reported for adults and children in CO-CSA households.

Low-income households that participate in CO-CSAs may be ‘positive deviants’ with respect to FVI. Positive deviants are at-risk members of a population whose uncommon practices enable them to achieve better outcomes than their peers who share similar risks [58]. In this study, adults and children from low-income households are known to be at-risk for low FVI but met recommendations for vegetable intake more often than average. The interpretation of CO-CSA members as positive deviants is consistent with evidence from a prior study that reported some CSA members ‘viewed themselves as already being heavy consumers of fresh vegetables and adventurous eaters’ and reported no change in vegetable consumption after joining a CSA [59]. The present study did not explore factors that enabled this group of low-income adults and children to more often meet recommendations for vegetable intake, but it is possible that CO-CSA participation may be one factor.

CO-CSAs warrant further examination as an avenue to improving vegetable consumption among adults and children in low-income households. A multi-state randomized controlled trial is currently underway to evaluate impacts of CO-CSA participation on FVI, food security, and related outcomes.

## Figures and Tables

**Table 1:** Characteristics of a cohort of 41 low-income households that participated in cost-offset community supported agriculture (CO-CSA).

	*N*	Count	%
**Household Characteristics**			
# household adults	41		
1 adult		9	22.0
2 adults		29	70.7
3+ adults		3	7.3
# household children	41		
1 child		10	24.4
2 children		18	43.9
3 children		8	19.5
4+ children		5	12.2
Food Security Status	39		
Food Secure		18	46.2
Low Food Security		14	35.9
Very Low Food Security		7	17.9
Food Assistance			
SNAP received in past month	39	20	51.3
WIC received in past month	39	16	41.0
Difficult to financially afford acceptable quality FV	39	21	53.8
Difficult to physically access acceptable quality FV	39	3	7.7
**Respondent Characteristics**			
Female	39	34	87.2
Age	39		
18–30 years		9	23.1
31–50 years		28	71.8
51+ years		2	5.1
Race	40		
White		35	87.5
Reported ‘Other’		5	12.5
Hispanic/Latino	39	2	5.1
Education	39		
High school or equivalent		4	10.3
Some college		8	20.5
College graduate		17	43.6
Graduate or professional degree		10	25.6
**Focal Child Characteristics**			
Female	41	20	48.8
Age	41		
2–3 years		8	19.5
4–8 years		18	43.9
9–12 years		15	36.6

Sample size varies by up to 2, due to missing data. SNAP, Supplemental Nutrition Assistance Program; WIC, Special Supplemental Nutrition Program for Women, Infants and Children; FV, fruits and vegetables.

**Table 2:** Total fruit and vegetable intake (FVI) in a cohort of 41 low-income households that participated in cost-offset community supported agriculture (CO-CSA).

	Median Intake, 2005 US MyPyramid Cup Equivalents			Met Recommendations from Dietary Guidelines for Americans, %		
	Sample	US		Sample	US ^c^	
Adults	(*n* = 38)	^a^		(*n* = 38)		
Fruit	0.97	0.52	**	18.4	18.4	
Vegetable	4.90	1.16	**	81.6	9.2	**
Children	(*n* = 41)	^b^		(*n* = 41)		
Fruit	1.25	0.85	**	51.2	49.9	
Vegetable	3.01	0.67	**	82.9	9.8	**

^a^ Original calculations to weight data from Moore, Dodd, Thompson et al. (2015) [8]. ^b^ Original calculations to weight data from Kim, Moore, Galuska, et al. (2014) [9]. ^c^ Original calculations to weight data from NCI (2015) [56]. Differences in medians were tested using Wilcoxon signed rank tests, and differences in percentages were tested using non-parametric binomial tests, with significance denoted: ** *p* < 0.01.

**Table 3:** Mean frequency of consumption of vegetables and fruits in a cohort of 41 low-income households that participated in cost-offset community supported agriculture (CO-CSA), by whether locally in-season or out-of-season.

	Adults (*n* = 23)			Children (*n* = 23)		
Vegetables	In-Season, Times/mo.	Out-of-Season, Times/mo.		In-Season, Times/mo.	Out-of-Season, Times/mo.	
Broccoli	6.4	10.5	*	4.3	5.5	
Cauliflower	3.2	7.4		1.7	3.7	**
Celeriac	0.8	0.5		0.2	0.3	^a^
Celery	3.7	6.9		3.3	5.5	
Corn (sweet)	3.5	3.1		4.6	2.4	*
Cucumbers	11.1	3.8	**	16.9	6.6	*
Eggplant	2.1	0.8	*	1.4	0.6	
Fennel	1.0	0.5		0.4	0.2	^a^
Beans (green, string, etc.)	10.5	4.8		7.5	4.0	
Parsnips	1.6	1.8		1.6	1.4	
Peas (snap, snow, and shell)	11.3	6.7	*	7.0	6.5	
Peppers	11.3	8.6		9.1	8.3	
Rhubarb	0.6	0.1	^a^	0.8	0.0	^a^
Squash, summer/zucchini)	7.2	2.6	***	5.0	1.6	**
Sweet potatoes	6.3	3.3		3.0	3.6	
Tomatoes	18.5	6.6	***	17.2	6.8	***
**Fruits**						
Apricots	1.5	1.3		1.4	0.7	
Blackberries	4.0	4.5		6.3	1.9	
Blueberries	17.3	6.2	**	15.7	6.3	**
Cherries	3.8	1.4	**	4.1	1.2	*
Currants	2.2	0.6	^a^	1.2	0.1	^a^
Elderberries	2.4	3.4		2.2	0.9	^a^
Gooseberries	1.3	0.2	^a^	0.6	0.0	^a^
Nectarines	5.1	1.8		4.9	0.6	*
Peaches	7.5	2.0	*	8.4	0.9	**
Pears	3.1	2.5		3.4	3.1	
Plums	4.1	2.7		3.7	0.9	**
Raspberries	9.9	6.5		11.5	5.1	*

^a^ Significance tests were not reported when fewer than five respondents reported a frequency >0. Differences were tested using paired samples t-tests, with significance denoted: * *p* < 0.05; ** *p* < 0.01; and *** *p* < 0.001. mo., month.
